# Users’ Perspectives on mHealth Self-Management of Bipolar Disorder: Qualitative Focus Group Study

**DOI:** 10.2196/mhealth.9529

**Published:** 2018-05-02

**Authors:** Lise Switsers, Arthur Dauwe, Anneleen Vanhoudt, Hilde Van Dyck, Koen Lombaerts, JFE Oldenburg

**Affiliations:** ^1^ Department of Educational Sciences Vrije Universiteit Brussel Brussels Belgium; ^2^ Faculty of Medicine and Health Sciences University of Antwerp Antwerp Belgium; ^3^ Board of Innovation Antwerp Belgium; ^4^ House of Innovation NV Antwerp Belgium; ^5^ Collaborative Antwerp Psychiatric Research Institute Faculty of Medicine University of Antwerp Antwerp Belgium; ^6^ Psychiatric Hospital Duffel University Department Duffel Belgium; ^7^ Curio NV Antwerp Belgium

**Keywords:** bipolar disorder, self-management, mHealth, focus groups

## Abstract

**Background:**

Recent research indicates that current mHealth apps for bipolar disorders (BDs) show crucial shortcomings. They lack important functionality, are of inconsistent quality, and are insufficiently evidence-based. mHealth apps need to be better adapted to the needs of users. The perspectives of adult service users with BD regarding mHealth apps have not been well investigated.

**Objective:**

The objective of this study was to examine the needs and expectations of adults with BD regarding mHealth apps.

**Methods:**

Two focus group sessions were organized in which patients’ views on self-management and design and functionality of an mHealth app for BD were assessed. During session 1, four focus groups were organized to identify users’ needs regarding support for self-management. Session 2 contained three cocreation focus groups. Through this method, the desired functionality and design were explored.

**Results:**

Participants indicated that they were in need of support in various ways. Not only support in psychoeducation, including daily routine, sleep pattern, maintaining social contacts, maintaining a healthy lifestyle, and avoidance of stimuli, was considered important for them but also gaining insight into their illness was found to be crucial.

**Conclusions:**

According to the participants, their illness-related information is a key factor in gaining insight into their mood pattern. Participants wanted a functional design that would increase daily use and prevent overstimulation. The results of this study should be taken into account when developing new mHealth apps.

## Introduction

Bipolar disorder (BD) is characterized by severe episodic disruption of mood. Medication alone is insufficient in reducing its impact [1]. Acquiring insight and monitoring of the illness are important for an optimal quality of life [2]. A crucial aspect is self-management [3]. Barlow et al [3] describe self-management as the individual’s capacity to cope with symptoms, treatment, potential physical and psychosocial effects, and lifestyle changes that are inextricably linked to living with a chronic illness. Thanks to effective self-management, patients can engage in suitable cognitive and emotional response, as well as handle the challenges related to their mental health to maintain a good quality of life [3].

The use of mobile computing and mobile communication technologies is growing rapidly in health care [4,5]. mHealth has great potential in the transformation of health care to improve its quality and efficiency [6]. It also has the ability to reduce costs in health care and improve its accessibility [7]. Mobile apps are a way to inform patients about their treatment process. As such, mHealth has the potential to empower patients. They can learn to manage their health more actively and independently through self-management [8,9]. mHealth apps, because of their usability, functionality, and proximity, are ideally suited to optimize the self-management of chronic diseases [9]. However, few mHealth apps for BD are evidence-based, most of them need to be better tailored to the needs and perspectives of people with BD [10,11], and user involvement remains a challenge [12,13]. Although intrinsic motivation of patients to learn is crucial for self-management and adherence [14], the current mHealth market has failed to develop an app that is both scientifically relevant and motivating [15].

For the development of evidence-based apps, it is important that developers take the needs of people with BDs into account [10-13], especially regarding the implementation of self-management strategies [5].

The qualitative studies among mHealth and especially user’s needs are very limited [16]. It should be noted that literature and research on the needs of people with BD and self-management strategies are lacking [17]. A study by Todd et al [18] focuses qualitatively on the needs of people with BD according to a Web-based self-management intervention. Other recent studies are researching quantitatively the needs of self-management apps [12] and focusing on the individual’s needs and opinions concerning existing apps or tools [13]. Therefore, this study is one of the first that aims to examine from scratch and broadly the needs and perspectives of people with BD concerning the development of a self-management app. By adding aspects of context mapping and cocreation, this research explores which elements of design and functionality should be included in a self-management app.

## Methods

In this qualitative study, a total number of 16 participants were represented, comprising 9 women and 7 men. These participants took part in the first 4 focus groups in session 1. Of this group of 16 participants, 10 participated a second time in the following 3 focus groups in session 2.

### Participants

To be eligible, participants had to report that they received a diagnosis of BD from a health professional, received a treatment for their BD or were undergoing treatment at the time of the study, and were aged at least 18 years. Another inclusion criterion consisted of a positive screening according to the Mood Disorder Questionnaire [19]. Participants were recruited through the Flemish patient network, Ups and Downs, a self-help organization for people suffering from BD. The average age was 42 years (SD 14) and the age range was 21 to 69 years. To investigate the subjective severity of their condition, participants were asked to indicate the severity of their BD on a scale (cf [18]) of 1 to 5, where 1 indicated the least severe, and a score of 5 indicated extreme severity. The average severity score of the participants was 3.5, indicating a moderate self-reported severity of BD. Sample characteristics are outlined in Table 1.

### Procedure

Seven focus groups were assembled with users with BD. Four groups took part in session 1, and 3 groups in session 2. In session 1, context mapping was used to gather insights into the users’ needs regarding self-management in general and mHealth self-management for BD specifically. Context mapping is a creative, exploratory research method, aimed at bringing out more latent needs and ideas [20].

Session 2 took place 10 weeks after session 1 and used cocreation to assess information about the users’ needs concerning the functionality and design of a self-management app. Cocreation is a form of collaboration in which participants are involved in the process of, in this case, designing a self-management app [21,22]. Both focus group sessions were 4 hours in length, including breaks of about 45 min in total. Audio and video data were collected during each session. The focus groups were conducted by trained clinicians under the supervision of the lead researchers. Due to the strong emotional impact of the subject and the intensity of the research methodology, each focus group had no more than 4 participants [23]. Four participants are sufficient for a group discussion and small enough for the moderator to pay attention to the input of each individual [24,25].

**Table 1:** Sample characteristics.

ID	Gender	Age, in years	Employed	Highest educational degree	Use of digital tools	Participation session 2	Severity score of bipolar disorder from 1 to 5
1	Female	59	No	Primary education	Computer, tablet, smartphone	Yes	3
2	Female	56	No	University education	Computer, mobile phone	Yes	5
3	Female	54	No	Higher education	Computer, smartphone	No	3
4	Female	47	No	Primary education	Computer, tablet, mobile phone	Yes	4
5	Female	37	Yes	Secondary education	Computer, tablet, smartphone	Yes	4
6	Female	24	No	Secondary education	Computer, tablet, smartphone	Yes	4
7	Female	36	No	University education	Computer, tablet, smartphone	No	3
8	Female	37	No	Higher education	Computer, tablet, smartphone	Yes	4
9	Female	21	No	Secondary education	Computer, smartphone	No	3
10	Male	36	No	Secondary education	Computer, tablet, smartphone	No	4
11	Male	28	No	Secondary education	Computer, tablet, smartphone	No	3
12	Male	41	No	Secondary education	Computer, tablet, smartphone	Yes	3
13	Male	53	No	University education	Computer, tablet, smartphone	No	2
14	Male	29	No	Secondary education	Computer, mobile phone	Yes	4
15	Male	69	No	University education	Computer, mobile phone	Yes	4
16	Male	41	Yes	Primary education	Computer, smartphone	Yes	4

### Session 1: Context Mapping

**Figure 1:**
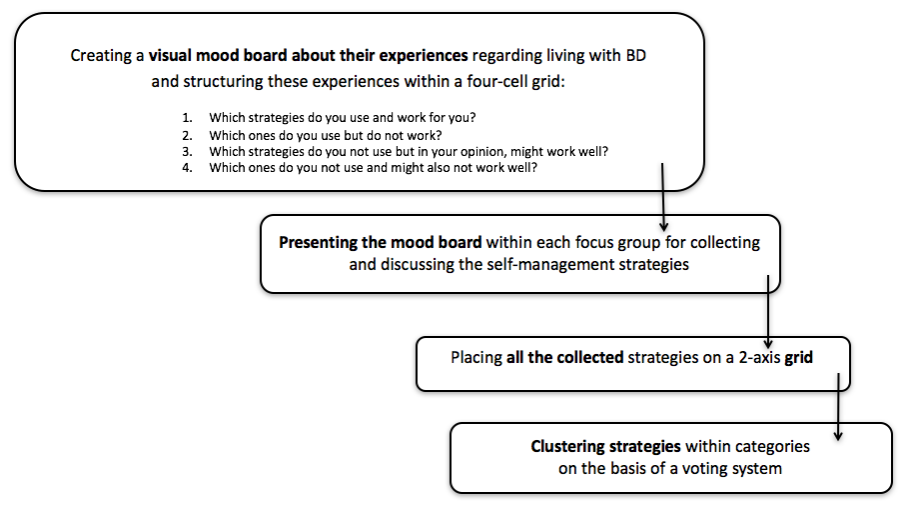
Flowchart on context mapping. BD: bipolar disorder.

In session 1, participants were asked about their experiences and needs regarding the self-management of their BD. Participants were invited to think creatively about the development and use of the app. Several creative exercises were presented to participants (Figure 1). They were first asked to create a mood board in the form of a scrapbook of magazine clippings about (potential) beneficial and harmful self-management strategies. Participants had to structure their scrapbook according to a 4-cell grid:

1. Which strategies do you use and work for you?
2. Which ones do you use but do not work?
3. Which strategies do you not use but in your opinion, might work well?
4. Which ones do you not use and might also not work well?

Structuring the exercise this way allowed us to gather information about positive and negative experiences participants have had with different self-management strategies. Participants were then asked to present their personal scrapbook to their focus group. Meanwhile, the moderator asked questions in case clarification was needed and collected the strategies that the participants found important. The third exercise was a brainstorming session. Participants had to write their preferred self-management strategies on post-it notes, selected from the strategies collected in the scrapbook exercise, and post them on a 2-axis grid. The 2 axes represented how pleasant versus how effective each strategy was considered. Subsequently, each participant indicated his or her 3 most important self-management strategies. These chosen strategies were then clustered allowing 4 main strategies to be chosen by the entire group of participants, the 4 focus groups.

### Session 2: Cocreation

Cocreation is a creative, explorative research method. This session was moderated according to various creative techniques. During the first exercise in the cocreation session, questions in the form of scenarios were asked and discussed within the 3 focus groups. These questions were based on the categories of the specific user’s needs gathered in session 1. The main categories form session 1 are love and relationships, psychological support, knowledge about yourself and disorder, rest, and avoidance of stimuli (examples of scenarios: You experience a crisis; how would you explain this to the app? How can the app possibly send a signal to the emergency services, family, or confidant? You want that you get enough sleep; How can the app facilitate this?) Respondents first had time to think about these scenarios individually, after which there was a discussion within the 3 different focus groups. Next, each focus group had to design an advertisement for a self-management app. The following elements were keys: a name, a commercial slogan, their target users, the main self-management strategy, one image, and the suggested price. This exercise allowed participants to think about the app in a different and creative way by making e-prototype of a self-management app; in addition, they needed to think about the essence of such an app. Participants of the focus groups were allowed to add further explanation if they thought it necessary. Thereafter, participants from the various focus groups presented their advertisements with accompanying explanations to the entire group of participants. Finally, each focus group had to design and draw screens for a possible self-management app on post-its. Subsequently, they presented their app to the entire group. During the presentations, questions and comments were posed by the entire group of participants.

### Analysis

During the focus groups, participants were encouraged to provide personal input. Although this produced very rich data, it presented challenges when attempting to align the needs and expectations of the participants. It was therefore key to look for factors that were important to all participants while still leaving room for individual creativity [26]. The qualitative data were submitted to both deductive and inductive analyses [27]. The analysis had a deductive aspect, as labels were derived from previous research focusing on BD and self-management apps [12,13,18]. Furthermore, the analyses included an inductive aspect, as new themes and labels were generated from the reading and analysis.

Focus groups were audio-recorded and transcribed verbatim. After the transcription, the first author saturated into the data by reading them several times with reference to the research questions. On the basis of the literature and feedback from the focus groups, an analysis scheme was created: the data were divided into fragments, tagged, organized, and categorized according to the open coding method, a technique that increases the chance of obtaining new insights [26]. Labels were assigned to the transcribed focus group data. For this purpose, we used the MAXQDA software (VERBI GmbH) to analyze the focus group data [26]. Labeling is assigning a name or code to similar phrases. A code shows the relationship between the words of the participants and the research questions. To facilitate this process, a labeling scheme was devised in which each core label was listed with associated sublabels [26,28]. The first author primarily completed the analysis, but feedback was derived from the coauthors to deepen the analyses. If coauthors disagreed on assigned labels, we reexamined this labeling to reach consensus. The description of the results is based on this labeling process.

### Ethics

The participants gave their oral and written informed consent before the start of each focus group. Ethical approval was granted by the Independent Ethics Committee (Committee for Medical Ethics at the University Hospital Antwerp), after consultation with the Ethical Committee of Emmaüs vzw (record no.: 15/13/128), Belgian registration number: B300201524839.

## Results

Data from the two sessions were analyzed together because aspects of design and functionality are closely interconnected. From sessions 1 and 2, four key themes were distilled from the data: (1) psychoeducation, (2) avoidance of stimuli, (3) social connection, and (4) personal support.

### Theme 1: Psychoeducation

Psychoeducation covered the following topics: insight into personal illness, information, medication, and therapy. These themes were selected by cross-referencing the data from the focus groups with an extensive literature review about the relationship between self-management strategies, triggers, and prodromes.

#### Insight Into Personal Illness

All participants indicated that their understanding of their BD is extremely important in improving self-management. Participants clearly expressed a need for support to gain insight into their disorder. For example, participants suggested that greater knowledge regarding their disease might aid early detection, which could encourage them to seek help sooner. Participants indicated that keeping a mood diary is useful to get a better understanding of their bipolar condition. Several participants already used a mood diary in a paper version, and some had experience with a digital version. Although most found it to be a very useful tool, they indicated that it was challenging to keep a diary at regular intervals. Participants claimed to be more committed when caregivers urged them to keep a diary. In the absence of such external motivation, most participants stopped keeping a journal even despite their belief that they could gain better control over their condition.

Importantly, participants expressed that one of the most powerful ways to gain insight is through gaining a better understanding of their episodic characteristics. Some indicated that the app could act as a “mirror” of their behavior. This means that they thought it would be a helpful feature of the app and provide knowledge and early warning signs regarding their prodromal behavior. In other words, they would like the app to “show” them their own behavior like a mirror would. One participant stated:

Everyone experiences a bipolar disorder differently; their effects are very individual. That’s what I notice within my circle of friends with bipolar disorder.

Participant 6, female, 24 years

Another participant indicated that she found it challenging to describe how she feels during a mood episode. Describing her daily activities is more tangible and concrete and would allow her to gain more insight into the course of her illness. She said:

When I start a manic episode for example, I spend a lot of money. I cannot describe my feelings during such a mood switch, but I can describe my expenses.

Participant 4, female, 47 years

In summary, participants stated that support for self-monitoring based on their behavior would be especially helpful for them to manage their mood.

#### Information

Another theme in psychoeducation is the acquisition of illness-related information. Participants indicated a need for information about medication, available assistance, and the course of their disorder. They felt insufficiently informed about available assistance and were often unaware of where they could find the right information. One participant felt that he was often sent from one care provider to another and could not get suitable support. Another participant indicated that she learned a lot by reading about BD, and this helped her to manage her condition more effectively:

[it is] important and really helps me to learn about bipolar disorder and read about stuff.

Participant 6, female, 24 years

According to participants, an app could be useful for accessing the correct information when it is needed.

#### Medication and Therapy

All participants took daily medication. They reported no moral problems to take their medication consistently and regularly. However, daily intake was difficult for some participants because of a lack of structure and daily routine. Participants suggested that it would be useful when the app can send a reminder or daily encouragement.

Therapy via a mobile self-management app was not a prominent topic mentioned during group discussions. Participants felt that face-to-face contact with a psychologist or therapist was very important; however, some indicated that therapy within a mobile self-management app had the potential to provide added value and support (eg, ability to contact social support, therapist, or psychologist quickly and more easily).

### Theme 2: Avoidance of Stress

#### Daily Routine and Good Sleep Hygiene

Participants frequently cited the importance of routine and structure for getting enough rest and sleep. Daily routine and good sleep hygiene” was an aspect whereby they need support. Participants found it challenging to maintain regular sleep and wake times. One participant commented:

Sleeping is very effective and useful, sleeping too much is not good either. To structure it, yes a good balance, is not easy.

Participant 11, male, 28 years

Good sleep hygiene was viewed as necessary, but many participants experienced challenges in this area.

One participant suggested that a mobile self-management app would be helpful if it could send her a message with a warning if she had lost sleep for a certain number of nights in a row. This would allow her to adjust her activities for the next few days as part of a preventive approach for mood episodes. Participants indicated that structure was important in their daily activities. Although they felt that an agenda could help, there was some ambivalence around this idea. One of the participants said:

Schemes and schedules to follow, it would certainly help, but it does not work. That currently interferes most in my daily life.

Participant 6, female 24 years

Some participants indicated that the main function of the app should focus on supporting them in maintaining structure in their lives.

#### Avoidance of Stimuli

A further topic that was extensively discussed in the focus groups relates to the avoidance of stimuli and stress. Participants indicated that the Internet, social media, smartphones, and tablets increase stimulation and are thus very distracting. Consequences included disrupted sleeping patterns, leading to feeling fatigued and having less structure in daily activities. A possible function of an app according to the participants would be to engage them in self-management but at the same time support them in taking time to rest. One participant indicated that if the mobile app could turn off daily between certain hours, it would avoid unnecessary stimulation and distraction:

The app switched off between certain hours, just quiet.

Participant 12, male, 41 years

Another participant suggested that the mobile app could suggest taking time out to rest. Interestingly, this aspect of avoiding stimuli is closely associated with the functionality of the app itself. Namely, the app itself was seen as a form of stimulus that should be minimized so as not to be invasive. Participants emphasized the importance of having a mobile app that would not distract too much or motivate them to use it continuously. Most participants indicated that pressure and stress had a significant negative impact on their mood; thus, an important self-management strategy should reduce stimulation. One participant stated:

I try to avoid business and stress and chaos.

Participant 5, female, 37 years

#### Relaxation

Participants stated that exercise as a relaxation strategy (eg, walking sports) was helpful for their mental state as well as improving self-management overall. Some participants suggested that an app could help to motivate them to perform these activities because they indicated that is difficult to do so regularly. However, personal preferences and individual factors are important to consider. Specifically, certain activities can be helpful for one individual, yet act as a trigger for another. For example, one participant indicated that sport could be a triggering factor for a manic episode. Another respondent indicated that mindfulness, relaxation, and breathing exercises accompanied by music would be calming and motivating for him, whereas others indicated such strategies would not be pleasant or useful for them. Mobile self-management apps need to take these nuances into account, supporting individuals to engage in activities that they not only enjoy but are also helpful for self-management of mood.

### Theme 3: Social Connection

Participants reported that both social relationships and contact with peers are important self-management strategies. Contact with others to exchange experiences, information, and tips was discussed as particularly helpful. They stated that maintaining social relationships also has an important supportive function. One participant said:

To me, good relations are very important for support. But if it goes wrong in a relationship, that is a trigger, then yes it could go wrong.

Participant 6, female 24 years

All participants agreed that an important function of the app would be to encourage them in supporting their social relationships. For example, the app could keep their friends and family informed on their well-being and contact the relevant person easily if the individual was in need of support. One participant considered this as potentially the core task of a self-management app:

...I want to let others know when I’m not well, the app would help me.

Participant 12, male, 41 years

Here, “ownership” was an important facet of the discussion. Some felt that family or friends should not be informed of their condition without their consent:

I would like to have control of the app. I would not like it if 5 people would automatically be notified.

Participant 8, female, 37 years

Thus, participants indicated that they should be able to decide when and who would receive information about them through the app.

### Theme 4: Personalized Support

Participants highlighted the importance of being able to customize an app, as this reflects the personalized nature of their BD and how they engage in self-management. One participant stated:

This works for me, but therefore not for anyone else. It’s all very personal.

Participant 8, female, 37 years

Being able to add a personal touch to the app would increase engagement and overall self-management efficiency. Participants indicated an important aspect here would be for the app to make suggestions, for example, supporting individuals by offering tailored information and suggestions for the management of their disorder. One of the participants said:

The app should be my life-coach.

Participant 5, female, 37 years

When asked to design an app, almost all participants indicated the importance of a crisis or emergency button in the app. This can again be personally programmed to either contact professional help, family or friends, or allow quick access to an automatic contingency plan.

Furthermore, participants mentioned that when the app is used in concert with health care workers, they would be more motivated to use the app. Participants found that the app should give tips and information using a positive and encouraging tone:

The text of the alert should be an encouring, positive, and interactive one. Not saying “watch out” but positive approach, more along the lines of: “I've noticed that...”

Participant 8, female, 37 years

Importantly, participants wanted control over the number of feedbacks generated by the app. This held for the number of tips, warning signals, and reminders that would suggest them to actively input data into the instrument. Therefore, a customizable setting would be preferable. Simple language was preferred, as participants felt that the use of the app would be adversely impacted by needlessly complex language. Visually, participants indicated a preference for the app to be appealing: playful, pleasant to look at, but simple. Finally, entering games or game elements into the app was barely mentioned during the focus groups although past research has shown that game elements can be useful to encourage health behavior [29,30]. Participants indicated that it is still a care-related app that must be taken seriously. On the other hand, small, limited gameplay elements might be applied to motivate them as long as they do not strongly distract them.

## Discussion

### Principal Findings

Participants highlighted their need for support in adopting various self-management strategies. The following self-management strategies were cited frequently: sleep, rest/relaxation, eating, exercise, self-monitoring, education about BD, relationships with others and finally, and a contingency plan. These results are consistent with previous patient-centered studies on successful self-management strategies in BD [31-33]. Research by Bair et al [34] shows several obstacles with regard to the use of self-management strategies, such as shortage of knowledge about strategies, absence of reliance on social support from family and friends, scarceness of medical communication, lack of time, or other priorities. These obstacles were found in our study as well. Any app should be adaptable to individual’s needs, so it can take such obstacles into account when providing support.

Congruent with previous research by Aujoulat et al [2], we found that the development of insight into illness was of great importance to users. Crucially, participants indicated a particular need to increase their understanding of their BD and state of mind by reflecting on their behavior. Prodromal behavior is more tangible than abstract mood values and thus gives users more foothold in early detection of possible episodes and subsequent strategic intervention. Gaining insight into which individual behavior accurately predicts mood destabilization is very important, and any self-management app should certainly support this.

Participants expressed their need for a coaching style to help them gain insight into their mood through their behavior. Rather than a directive instructor, their preference was for an app to act more like a personal coach encouraging better understanding of their unique condition and gently guiding them toward insight and successful self-management—in addition, the way in which the app encourages the users’ needs to be tailored to the individual. The question then is, how is this aspect of coaching best integrated into a self-management app? Participants provided a few ideas here. One is that the app itself would be the coach who gives suggestions and acts as an educator. Conversely, coaching could be done in concert with a psychologist or psychiatrist. This corresponds to use in a blended care program. Blended care occurs when a (digital) stand-alone treatment such as internet-based therapy is supported by short face-to-face consultation with care providers such as a psychologist or psychiatrist [35]. This method of coaching could be integrated into the use of the self-management app. According to participants, if the self-management app were supported by professional assistance, it would encourage greater adherence, a finding that is supported in previous literature [36-38].

Participants indicated that the app should provide support for the use of various self-management strategies from which they can make a selection according to their personal needs. According to the participants, this malleability would benefit the sustainable usability of the app. These results are supported by Holmstöm and Roing [39] who state that quality of care increases when the patient is the owner of their own care process. This study shows that users have very differing needs and wants regarding a self-management app. As they indicate, prodromal and episodic symptoms can vary widely between individuals. In addition, strategies that work for one user might not work for another or might even adversely influence their mood state. This holds both for functionality and design elements of an app. Where some functions (eg, relaxation therapy, encouragement in exercise, playing restful music, etc.) are vital for one user, it might put off another to use the app. Although a truly individual app might not be feasible, it is clear that a one-size-fits-all approach is inappropriate. User preferences from this study indicate that those with BD should at least be able to maximally choose which features of the app they want to use and how to use them. These results are congruent with a recent study by Nicholas et al [12], indicating that young adults want a range of self-management strategies supported by different app functions.

Torous and Powell [40] indicate that a distinction can be made between active and passive apps. Active apps require practical cooperation of the patient, whereas passive apps do not. A passive app can have a diagnostic role and monitors symptoms, whereas an active app can provide interventions such as sending reminders regarding health. The results of this study suggest that participants would prefer both elements in a self-management app for BD. They require the choice between several guises of active or passive use (ie, a diagnostic role, reminders, and psychoeducation) on the basis of their personal behavior. Providing this choice might motivate users to engage with the app.

### Limitations

This study has a number of limitations. Although participant groups represented men and women of different ages, the generalizability of the study to individuals with BD is limited because of the qualitative nature of the findings. A further limitation of this study is the self-reported diagnosis of BD, rather than a clinical diagnosis.

Our sample was relatively homogeneous in the sense that most participants were recruited through a patient support group. These participants might be more actively engaged in managing their condition than others who are not members of such peer groups. The aspects of psychoeducation came up regularly during the group discussions. Importantly, acknowledging the mood disorder is crucial for psychoeducation to take effect [41]. We cannot determine how the level of illness awareness influenced the results. However, most of our participants took part in a peer-led support group. Therefore, they might have a higher level of illness awareness.

Most participants rated the severity of their BD as 3 or above on a 5-point scale. Individuals with lower severity ratings were not included in the sample; thus, it is unclear whether the perceived burden of the illness would affect study outcomes.

Finally, the majority of participants were unemployed. This may have resulted in an overestimation of the need for structure, which featured prominently in our results.

### Conclusions

There are limited studies exploring the needs of people with BD with respect to mHealth [12,18]. This research adds insight into the needs of people with BD regarding the use of self-management strategies and how these could best be supported by mHealth apps. Study findings aim to bridge the gap between research and development that is currently present in the mHealth market.

Individuals with BD in this study expressed a need for interventions that provide solutions, suggestions, ideas on overcoming barriers, and personalized support. In addition, psychoeducational components including building insight into their condition and behavior were viewed as crucial aids for self-management. Behavioral insights were deemed to be important predictors of mood dysregulation. Furthermore, participants indicated that the app should act as a personalized coach rather than a strict instructor.

Finally, although this study is an important step in the integration of user perspectives in the mHealth design for BD, future work with larger samples with varying characteristics is needed to ensure that mHealth developments take a wide variety of users’ needs and preferences into account. This, in turn, will provide users with powerful digital health care tools that empower them in managing their well-being and improve longer term outcomes.

## Abbreviations

BD: bipolar disorder
